# Why do Integrated Maternal HIV and Infant Healthcare Services work? A Secondary Analysis of a Randomised Controlled Trial in South Africa

**DOI:** 10.1007/s10461-023-04097-x

**Published:** 2023-06-12

**Authors:** Kirsty Brittain, Karryn Brown, Tamsin Phillips, Allison Zerbe, Jennifer Pellowski, Robert H. Remien, Claude A. Mellins, Elaine J. Abrams, Landon Myer

**Affiliations:** 1 Division of Epidemiology & Biostatistics, School of Public Health & Family Medicine, University of Cape Town, Anzio Road, Observatory, Cape Town, 7925 South Africa; 2 Mailman School of Public Health, ICAP at Columbia University, Columbia University, New York, NY USA; 3 Department of Behavioral & Social Sciences, Brown University School of Public Health, Providence, RI USA; 4 International Health Institute, Brown University School of Public Health, Providence, RI USA; 5 New York State Psychiatric Institute, HIV Center for Clinical & Behavioral Studies, Columbia University, New York, NY USA; 6 Department of Pediatrics, Vagelos College of Physicians & Surgeons, Columbia University, New York, NY USA; 7 Department of Epidemiology, Mailman School of Public Health, Columbia University, New York, NY USA

**Keywords:** Integration, Retention, PMTCT, Intervention, South Africa

## Abstract

In a randomised trial, we found that integrated maternal HIV and infant health services through the end of breastfeeding were significantly associated with the primary outcome of engagement in HIV care and viral suppression at 12 months postpartum, compared to the standard of care. Here, we quantitatively explore potential psychosocial modifiers and mediators of this association. Our findings suggest that the intervention was significantly more effective among women experiencing an unintended pregnancy but did not improve outcomes among women reporting risky alcohol use. Although not statistically significant, our results suggest that the intervention may also be more effective among women experiencing higher levels of poverty and HIV-related stigma. We observed no definitive mediator of the intervention effect, but women allocated to integrated services reported better relationships with their healthcare providers through 12 months postpartum. These findings point to high-risk groups that may benefit the most from integrated care, as well as groups for whom these benefits are hampered and that warrant further attention in intervention development and evaluation.

**Supplementary Information:**

The online version contains supplementary material available at 10.1007/s10461-023-04097-x.

## Introduction

Increases in the availability and uptake of antiretroviral therapy (ART), including under guidelines of universal ART for pregnant and breastfeeding women, have led to massive reductions in new paediatric HIV infections [1–3]. However, poor retention in ART services remains a global concern, with particularly high levels of disengagement from HIV care documented among women during the postpartum period [4–6]. Pooled data from Option B + programs in Africa suggest that approximately 75% of women are retained in HIV care through 6–12 months postpartum [5]. In South Africa and many similar settings, prevention of mother-to-child transmission (PMTCT) services are integrated into antenatal care during pregnancy, but the timing of transfer out of antenatal services during the postpartum period differs across countries [7]. In South Africa, this transfer occurs immediately postpartum. Under these policies, women receive HIV services separate from routine infant care which may be provided in different locations. Across and within countries, policies related to the timing of transfer may be implemented differently in different health facilities [8], and transfer out of antenatal services has been highlighted as a major point of loss to follow-up in the maternal HIV care cascade [2, 9].

An approach which has been posited to reduce disengagement from care is the integration of HIV care into other routine health services, although the current evidence for effectiveness is mixed [10, 11]. In settings where integrated services have been shown to be effective, there is a need to examine factors that potentially modify intervention effects, and to explore the mechanisms of change leading to improved outcomes. This evidence is particularly important in low-resource settings to refine interventions and potentially target them to particular groups for whom they are most beneficial. In the context of a randomised controlled trial in Cape Town, South Africa [the “Maternal and Child Health – Antiretroviral Therapy” (MCH-ART) study], we compared the existing standard of care (control) to an integrated service (intervention), where maternal ART and infant health care were provided concurrently and in the same maternal and child health (MCH) clinic through the end of breastfeeding. We found that providing integrated services significantly increased the proportion of women who were engaged in HIV care and virally suppressed at 12 months postpartum [12]. We posited that the effectiveness of the intervention overall may be due to factors which include a lower burden of clinic visits due to co-located care; lower levels of HIV-related stigma through continued receipt of ART in the MCH clinic; and prolonged positive relationships with nurse-midwives in this clinic [13–15]. Further, we observed that the intervention was more effective among women initiating ART later in pregnancy, compared to those initiating ART earlier [12].

Given the promise of this model of care for postpartum women, there is a need to refine and target future implementation of this intervention. Critical to this refinement is the identification of factors that may modify intervention effects, and an exploration of mechanisms of change. A framework that may be beneficial to this is Social Action Theory (SAT). SAT delineates a model of contextual determinants as well as social interaction and individual self-regulatory processes, and the mechanisms by which these lead to health behaviours [16, 17]. Contextual factors known to be associated with poor ART outcomes include an individual’s background characteristics such as poverty [18–20], and internal contextual factors include mental health problems such as depression or psychological distress [18, 21, 22] as well as alcohol use [19, 21–24]. Among pregnant women specifically, other contextual determinants include late entry into antenatal care [18–20] and an unintended pregnancy [25, 26]. Social interaction processes that may negatively affect ART outcomes include negatives attitudes from or treatment by healthcare providers [18–20], HIV-related stigma [18–20, 27, 28], low levels of social support [18, 19, 22], and the fear or experience of intimate partner violence (IPV) [19, 20]. Finally, factors known to be associated with suboptimal ART behaviours at the level of individual self-regulation include poor knowledge about HIV and ART [19, 20, 29, 30], negative beliefs and concerns about ART use [19, 20, 31, 32], and low levels of adherence-self efficacy [20, 21, 33].

Following the conclusion of the MCH-ART randomised controlled trial, our aim here was to quantitatively examine (i) psychosocial modifiers of the intervention effect and (ii) potential mediators of the effect. The goal of examining modifiers was to identify groups for whom the intervention was most beneficial and to whom the intervention could be targeted, as well as groups for whom the intervention was not effective and that warrant further attention in intervention development. In parallel, the goal of examining mediators was to understand how integrated services lead to improved outcomes, with a view to refining interventions by identifying the most effective components.

## Methods

This analysis draws on data from the MCH-ART trial, which evaluated integrated services during the postpartum period. The design and primary results of the trial have been previously reported [12, 34]. Briefly, the study was conducted in a primary care antenatal clinic in the community of Gugulethu, Cape Town, where an antenatal HIV prevalence of ~ 30% has been reported [35]. In this setting, women experience multiple overlapping risks, including poverty, unintended pregnancy, alcohol use, and IPV [36–38]. For the MCH-ART study, women living with HIV were enrolled when entering antenatal care, and women initiating ART were followed through delivery. Following enrolment, women attended up to three study measurement visits, coinciding with their 2^nd^ antenatal visit; late 3^rd^ trimester; and immediately postpartum.

### MCH-ART Intervention

Women who attended the immediate postpartum study visit and were currently breastfeeding [471 (75%) of 628 women enrolled into antenatal follow-up] were enrolled into postpartum follow-up and randomly allocated. Random allocation was to the MCH-ART intervention (integrated concurrent and co-located maternal ART and paediatric care in the MCH clinic through the end of breastfeeding) or the standard of care (control), where women were referred as soon as possible postpartum to general HIV clinics, and their infants were referred to routine child health services. Although all postpartum women may benefit from integrated HIV and MCH care, the goal of this trial was to focus on breastfeeding women, given the ongoing risk of mother-to-child transmission (MTCT). Thus, only breastfeeding women were enrolled into the trial; a secondary aim of the trial was to explore the impact of the intervention on breastfeeding practices. Women subsequently attended up to five study measurement visits separate from any routine health services, at 6 weeks and 3, 6, 9 and 12 months postpartum. This analysis includes all women with primary outcome data available at 12 months postpartum [411 (87%) of 471 women enrolled]. All participants provided written informed consent prior to study enrolment. The study was approved by the Faculty of Health Sciences Human Research Ethics Committee at the University of Cape Town, and the Institutional Review Board of Columbia University Medical Centre.

### Measures

Table 1 details the measures assessed at each study measurement visit, relevant to this analysis. All measures were administered by trained interviewers in participants’ home language, predominantly isiXhosa. Instruments were translated from English into isiXhosa and were back-translated using standard procedures [39]. Sociodemographic characteristics were assessed at enrolment, and a composite poverty score was created based on current employment status, type of housing (formal house or informal shack) and access to household assets (a flush toilet, piped water inside the home, electricity, a refrigerator, a telephone, and a television). Distribution-based cut-offs were used to categorize participants into three groups, representing the most disadvantaged (lowest scores), moderately disadvantaged (middle), and least disadvantaged women (highest scores) relative to other participants in the study based on this composite score. Women were asked using a single item whether they were trying to have a baby when they became pregnant, with pregnancy intentions categorised as planned versus unplanned.

**Table 1:** Factors explored as (A) effect modifiers and (B) mediators of the intervention effect, across timepoints of assessment

	Antenatal			Postpartum					
	1^st^ antenatal visit	2^nd^ antenatal visit	Late 3^rd^ trimester	< 7 days ***Random allocation to intervention versus control***	6 weeks	3 months	6 months	9 months	12 months ***Primary trial outcome***
Poverty	A								
Pregnancy intentions	A								
HIV knowledge		A			B			B	
HIV treatment knowledge		A			B			B	
ART medication beliefs		A			B			B	
Adherence self-efficacy		A	A				B		B
HIV-related stigma		A			B				B
Social support		A	A				B		B
Patient-provider relationship					B		B	B	B
Depression (EPDS)		A							
Psychological distress (K-10)		A							
Risky alcohol use (AUDIT-C)		A	A						
Intimate partner violence		A		A					

Abbreviations: EPDS: Edinburgh Postnatal Depression Scale; K-10: Kessler-10 scale; AUDIT-C: Alcohol Use Disorders Identification Test – Consumption.

At various antenatal and postpartum timepoints, a battery of psychosocial measures was administered. Measures were selected drawing on SAT, described above, and on factors known to be associated with adherence behaviours. We adapted previously used tools to assess HIV and HIV treatment knowledge [40–42], ART medication beliefs [43] and adherence self-efficacy [44]. Knowledge scores were summed, and we calculated mean scores for medication beliefs and self-efficacy; higher scores on each scale indicate higher levels of knowledge, beliefs about the necessity of taking ART, and adherence self-efficacy, respectively. We used items adapted from the Social Impact Scale to measure enacted and internalised HIV-related stigma [45], and from a measure of the perceived availability of social support to measure social support [46, 47]; both measures have been shown to perform well in this setting [48]. For these measures, higher scores indicate higher levels of HIV-related stigma and higher levels of perceived social support, respectively. Finally, women’s relationships with their routine healthcare providers were assessed using a 19-item Patient-Healthcare Provider Relationship Scale which was developed and validated among South African pregnant women [49], with higher scores indicating better patient-provider relationships. In analysis, we explored each of these constructs as continuous variables and using categories of above versus below the median score to classify participants according to relative levels.

Depression during the past week was assessed using the Edinburgh Postnatal Depression Scale (EPDS), which has been validated for use during pregnancy [50], with a score of ≥ 13 used to indicate possible depression [51]. Psychological distress during the past month was measured using the Kessler-10 (K-10) scale [52], with a threshold of ≥ 21.5 used to indicate psychological distress [53]. Alcohol use was measured using the Alcohol Use Disorders Identification Test - Consumption (AUDIT-C), with a score of ≥ 3 used to indicate risky drinking [54, 55]. At the 2^nd^ antenatal study visit, women were asked to report alcohol use during the 12 months prior to pregnancy recognition; at the study visit conducted during the late 3^rd^ trimester, women were asked about alcohol use during pregnancy. IPV was assessed using the World Health Organization Violence Against Women tool [56], with violence defined as any report of psychological, physical or sexual violence. Violence during the 12 months prior to pregnancy recognition was assessed at the 2^nd^ antenatal study visit, and violence during pregnancy was assessed immediately postpartum.

At study visits, women provided 5ml of venous blood for HIV viral load (VL) testing, conducted by the National Health Laboratory Service (NHLS) using the Abbott Molecular RealTime HIV-1 assay (Abbott Molecular, Illinois, USA). Data on health service usage was requested from the Western Cape Provincial Health Data Centre, including data pertaining to HIV care visits; pharmacy dispensing; and laboratory results. The primary outcome for the MCH-ART trial was a binary composite endpoint of retention in care and viral suppression at 12 months postpartum. Retention in care was defined as any evidence of HIV-related clinical care received between 9 and 18 months postpartum, based on routinely collected records of HIV clinical care visits, ART dispensing, and HIV-related laboratory testing. Viral suppression was defined as VL < 50 copies/ml at the 12 month postpartum study visit. Women were considered as having achieved the primary endpoint if they had both evidence of retention in care and VL < 50 copies/mL [12].

### Data Analysis

Data were analysed using STATA V14.2 (Stata Corporation, College Station, Texas, USA) and R (R Foundation for Statistical Computing, Vienna, Austria). To assess modifiers of the intervention effect, we used stratified χ^2^ analyses and regression models including interaction terms. We also calculated stratum-specific risk differences (RD) for each potential effect modifier using additive binomial models and report coefficients with 95% confidence intervals (CI). For these analyses of effect modification, we used data from study visits prior to randomisation. For measures that were assessed multiple times prior to randomisation, consistent results were observed using later measures, thus we report results using the first measure of each construct. Consistent results were also observed when using sub-scales of the psychosocial measures and when exploring the effect of different forms of IPV. To assess mediation, we explored associations between trial arm and potential mediators measured after randomisation; and calculated the percentage change in the RD corresponding to the intervention effect when each potential mediator was added to the model. All analyses were exploratory in nature, thus we did not correct for multiple comparisons or use confirmatory analytic techniques such as structural equation modelling [57]. Rather than relying solely on p-values to indicate statistical significance, we focus on effect sizes and 95% CI to indicate the precision of our findings [58].

## Results

Between March 2013 and April 2014, 628 pregnant women living with HIV were enrolled into antenatal follow-up. Of these women, 471 (75%) attended the immediate postpartum study visit and reported breastfeeding, and were enrolled into the MCH-ART trial. Compared to those enrolled, women who were excluded had entered antenatal care earlier (mean: 19.3 weeks versus 20.9 weeks; *z*: -2.36; p-value: 0.019) and had higher HIV treatment knowledge scores (mean score: 6.6 versus 6.5 out of a maximum score of 8; *z*: 1.93; p-value: 0.053) and higher social support scores (mean score: 4.4 versus 4.2 out of a maximum score of 5; *z*: 1.83; p-value: 0.068); no other demographic or psychosocial characteristics differed between women enrolled into the trial versus those not enrolled (Supplementary Table 1). Of the 471 women enrolled into the trial, 411 (87%) contributed primary outcome data (202 allocated to the intervention arm, and 209 to the control) and are included in this analysis. The mean age overall was 28.8 years; 25% had completed secondary education; and 38% were currently employed. Just over half of women (54%) were newly diagnosed HIV-positive; and 41% were married and/or cohabiting with their male partner. Baseline characteristics were similar across intervention and control, although women allocated to the control had entered antenatal care at a slightly later gestation compared to women allocated to the intervention (mean: 21.1 versus 19.9 weeks; *z*: -1.70; p-value: 0.090; Table 2).

**Table 2:** Baseline demographic characteristics and potential psychosocial modifiers of the intervention effect, by trial arm (intervention versus control)

	Total sample – Mean (SD)	Intervention – Mean (SD)	Control – Mean (SD)	χ^2^ or z statistic^1^	P-value
Number of participants	411	202	209		
Age in years	28.8 (5.5)	28.9 (5.3)	28.7 (5.6)	0.51	0.611
N (%) having completed secondary/any tertiary education	101 (25%)	45 (22%)	56 (27%)	1.13	0.288
N (%) currently employed	155 (38%)	79 (39%)	76 (36%)	0.33	0.566
N (%) married and/or cohabiting	169 (41%)	89 (44%)	80 (38%)	1.42	0.234
N (%) diagnosed HIV-positive during current pregnancy	223 (54%)	104 (51%)	119 (57%)	1.23	0.267
Gestation at entry into antenatal care (weeks)	20.5 (7.6)	19.9 (7.7)	21.1 (7.5)	-1.70	0.090
Poverty categories – n (%)					
Most disadvantaged	143 (35)	68 (34)	75 (36)		
Moderate disadvantage	137 (33)	76 (38)	61 (29)		
Least disadvantaged	131 (32)	58 (29)	73 (35)	3.58	0.167
N (%) reporting an unintended pregnancy	291 (71)	145 (72)	146 (70)	0.18	0.668
HIV knowledge score (max = 9; n = 409)	5.8 (1.3)	5.9 (1.3)	5.8 (1.4)	0.50	0.618
HIV treatment knowledge score (max = 8; n = 409)	6.5 (1.2)	6.6 (1.1)	6.4 (1.2)	2.32	0.020
ART medication beliefs score (max = 5; n = 409)	4.0 (0.7)	4.0 (0.7)	4.0 (0.7)	0.14	0.887
Adherence self-efficacy score – 2^nd^ antenatal visit (max = 5; n = 407)	4.8 (0.4)	4.8 (0.3)	4.7 (0.4)	2.30	0.021
Adherence self-efficacy score – late 3^rd^ trimester (max = 5; n = 405)	4.8 (0.4)	4.8 (0.4)	4.8 (0.4)	0.96	0.336
HIV-related stigma score (max = 5; n = 408)	2.2 (0.8)	2.2 (0.8)	2.2 (0.8)	0.62	0.536
Social support score – 2^nd^ antenatal visit (max = 5; n = 409)	4.2 (0.8)	4.2 (0.9)	4.2 (0.8)	0.72	0.472
Social support score – late 3^rd^ trimester (max = 5; n = 406)	4.3 (0.8)	4.3 (0.9)	4.3 (0.8)	0.50	0.618
N (%) scoring above threshold for depression (EPDS; n = 409)	41 (10)	16 (8)	25 (12)	1.78	0.182
N (%) scoring above threshold for psychological distress (K-10; n = 409)	26 (6)	9 (5)	17 (8)	2.27	0.132
N (%) scoring above threshold for risky drinking – 2^nd^ antenatal visit (AUDIT-C; n = 409)	105 (26)	50 (25)	55 (26)	0.09	0.761
N (%) scoring above threshold for risky drinking – late 3^rd^ trimester (AUDIT-C; n = 406)	38 (9)	19 (10)	19 (9)	0.04	0.848
N (%) reporting any intimate partner violence – 2^nd^ antenatal visit (n = 407)	93 (23)	43 (22)	50 (24)	0.41	0.524
N (%) reporting any intimate partner violence – <7 days postpartum (n = 411)	27 (7)	14 (7)	13 (6)	0.08	0.771

Abbreviations: SD: standard deviation; EPDS: Edinburgh Postnatal Depression Scale; K-10: Kessler-10 scale; AUDIT-C: Alcohol Use Disorders Identification Test – Consumption. All measures are from the 2^nd^ antenatal visit, unless otherwise specified. ^1^ Statistic from χ^2^ or Wilcoxon rank-sum test.

### Modifiers of the Intervention Effect

**Fig. 1:**
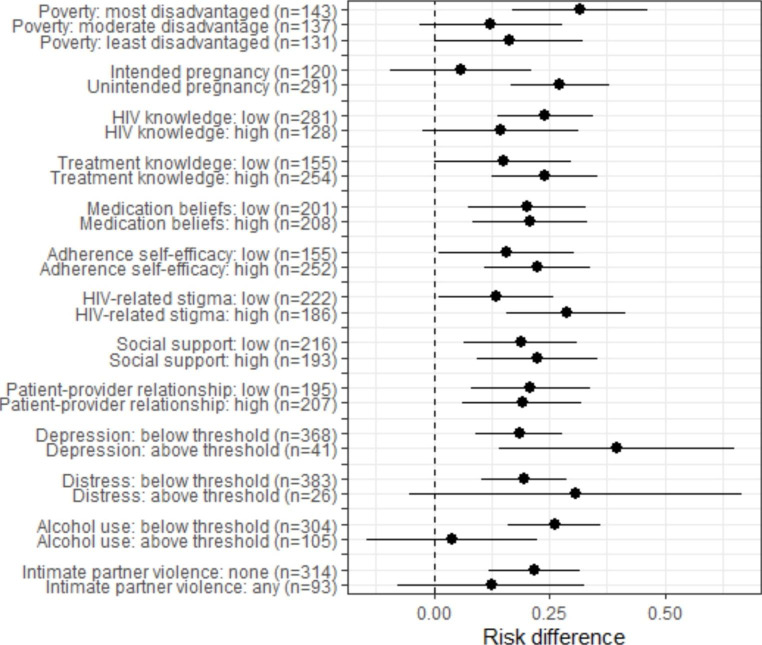
Forest plot of primary outcome across subgroups of psychosocial characteristics measured prior to randomisation

Psychosocial characteristics relevant to the analyses of effect modification are summarised in Table 2. These measures were administered prior to randomisation. Overall, we observed high levels of unintended pregnancy, risky alcohol use and IPV; high levels of adherence self-efficacy and social support; and low levels of HIV and treatment knowledge. No psychosocial characteristics differed appreciably across the intervention versus control group. In the overall sample, the RD for the primary outcome between intervention and control was 0.21 [95% CI: 0.12, 0.30]. We observed no evidence of effect modification by HIV or treatment knowledge; ART medication beliefs; adherence-self efficacy; social support; or patient-provider relationships during the antenatal period (Fig. 1; Table 3). In addition, depression, psychological distress, and IPV did not modify the intervention effect.

**Table 3:** Intervention effect, stratified by potential effect modifiers

	Risk difference	P-value for interaction
	[95% confidence interval]	
**Overall intervention effect**	**0.21 [0.12, 0.30]**	
Poverty		
Most disadvantaged	0.32 [0.17, 0.46]	
Moderate disadvantage	0.12 [-0.03, 0.28]	0.077
Least disadvantaged	0.16 [0.001, 0.32]	0.17
Pregnancy intentions		
Intended pregnancy	0.06 [-0.09, 0.21]	
Unintended pregnancy	0.27 [0.17, 0.38]	0.025
HIV knowledge score		
Low	0.24 [0.14, 0.34]	
High	0.15 [-0.02, 0.31]	0.343
HIV treatment knowledge score		
Low	0.15 [0.002, 0.30]	
High	0.24 [0.13, 0.35]	0.342
ART medication beliefs score		
Low	0.20 [0.07, 0.33]	
High	0.21 [0.08, 0.33]	0.948
Adherence self-efficacy score		
Low	0.16 [0.01, 0.31]	
High	0.23 [0.11, 0.34]	0.48
HIV-related stigma score		
Low	0.13 [0.01, 0.26]	
High	0.29 [0.16, 0.42]	0.094
Social support score		
Low	0.19 [0.07, 0.31]	
High	0.23 [0.09, 0.36]	0.688
Patient-provider relationship score		
Low	0.21 [0.08, 0.34]	
High	0.19 [0.06, 0.32]	0.842
Depression (EPDS)		
Below threshold	0.18 [0.09, 0.28]	
Above threshold	0.40 [0.14, 0.65]	0.129
Psychological distress (K-10)		
Below threshold	0.20 [0.10, 0.29]	
Above threshold	0.31 [-0.05, 0.67]	0.561
Risky alcohol use (AUDIT-C)		
Below threshold	0.26 [0.16, 0.36]	
Above threshold	0.04 [-0.15, 0.23]	0.04
Intimate partner violence		
None	0.22 [0.12, 0.31]	
Any	0.12 [-0.08, 0.33]	0.422

Abbreviations: EPDS: Edinburgh Postnatal Depression Scale; K-10: Kessler-10 scale; AUDIT-C: Alcohol Use Disorders Identification Test – Consumption.

**Fig. 2:**
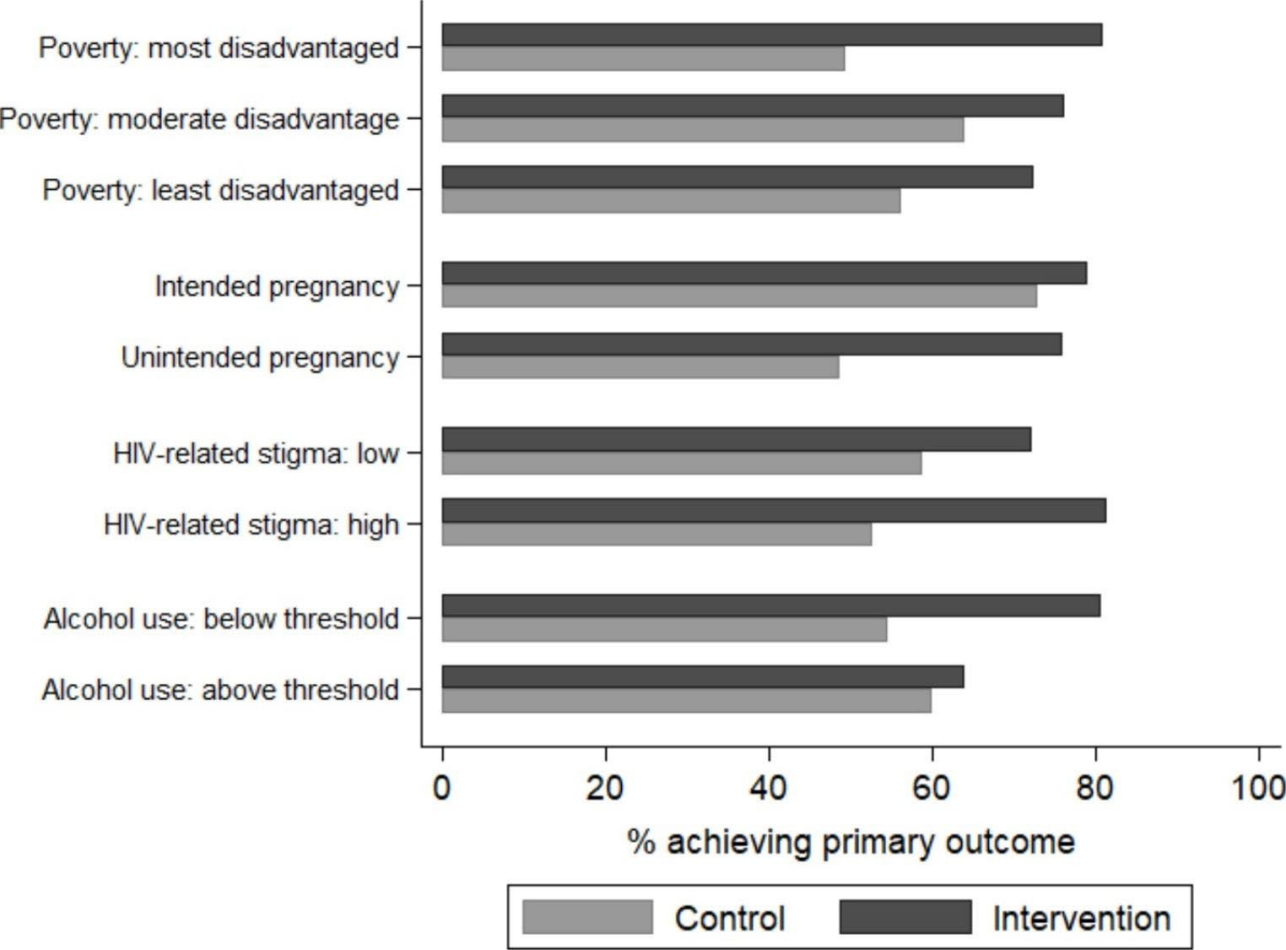
Proportion of women allocated to the intervention and control achieving the primary trial outcome (engagement in HIV care and viral suppression at 12 months postpartum) across subgroups of psychosocial characteristics measured prior to randomisation

However, there was some evidence that the intervention was more effective in particular groups of women, although not all differences were statistically significant (Fig. 1; Table 3). Figure 2 graphically illustrates these interaction effects by showing the proportion of women allocated to the intervention and control who achieved the primary trial outcome across groups. First, although not statistically significant, the intervention effect was stronger among women experiencing higher levels of poverty. Among women experiencing the highest levels of disadvantage, 81% of those in the intervention arm achieved the trial outcome, compared to 49% of those in the control (RD: 0.31; 95% CI: 0.17, 0.46). In contrast, 76% and 64% of women experiencing moderate levels of disadvantage and allocated to the intervention and control, respectively, achieved the trial outcome (RD: 0.12; 95% CI: -0.03, 0.28; p-value for interaction: 0.077). Among women experiencing the lowest levels of disadvantage and allocated to the intervention and control, 72% and 56% achieved the trial outcome, respectively (RD: 0.16; 95% CI: 0.001, 0.32; p-value for interaction: 0.170). Second, although also not statistically significant, the intervention effect was stronger among women reporting higher levels of HIV-related stigma. Among women reporting high levels of stigma, 81% of those in the intervention arm achieved the trial outcome, versus 53% of those in the control (RD: 0.29; 95% CI: 0.16, 0.41). In comparison, 72% and 59% of women reporting low levels of stigma and allocated to the intervention and control achieved the trial outcome, respectively (RD: 0.13; 95% CI: 0.01, 0.26; p-value for interaction: 0.094).

In addition, the intervention effect differed significantly according to two characteristics: pregnancy intention and alcohol use. Among women reporting that their pregnancy was unintended, 76% of women in the intervention arm achieved the trial outcome, versus 49% of those in the control (RD: 0.27; 95% CI: 0.16, 0.38). In comparison, 79% and 73% of those reporting that their pregnancy was intended and allocated to the intervention and control achieved the trial outcome, respectively (RD: 0.06; 95% CI: -0.09, 0.21; p-value for interaction: 0.025). Finally, women who reported risky drinking in the 12 months prior to pregnancy recognition were significantly less likely to benefit from the intervention. In this group of women, 64% of those in the intervention arm and 60% of those in the control achieved the trial outcome (RD: 0.04; 95% CI: -0.15, 0.23), compared to 81% and 55% among those not reporting risky drinking, respectively (RD: 0.26; 95% CI: 0.16, 0.36; p-value for interaction: 0.040).

### Mediators of the Intervention Effect

No appreciable differences were observed in HIV or treatment knowledge, ART medication beliefs or adherence self-efficacy between the intervention and control group at study measurement visits after randomisation (Table 4). Similarly, levels of HIV-related stigma and social support did not differ across groups over time. However, we observed a strong association between trial arm and postpartum patient-provider relationships, with women allocated to the intervention reporting significantly better relationships with their routine healthcare providers at 6, 9 and 12 months postpartum compared to women allocated to the control (Table 4). When each potential mediator was added to a model of the intervention effect, the percentage change in the RD for the intervention effect was negligible across potential mediators, ranging from − 2.1 to 4.3% (Table 4). When examining the effect of variables measured after randomisation on the primary outcome (combined binary endpoint of retention in HIV care and viral suppression), few significant associations were observed (Table 5). Women who achieved the primary outcome had slightly higher adherence self-efficacy and social support scores at 6 months postpartum, but these associations were not sustained at 12 months postpartum. As reported above, women allocated to the intervention reported significantly better relationships with their healthcare providers compared to those allocated to the control, but patient-provider relationships were not associated with the primary outcome.

**Table 4:** Potential postpartum mediators of the intervention effect, by trial arm (intervention versus control), and impact of potential mediators on the intervention effect

	Comparison of mediators by trial arm					Impact of mediators on intervention effect	
	Total sample – Mean (SD)	Intervention – Mean (SD)	Control – Mean (SD)	z statistic^1^	p-value	Risk difference [95% CI]	Percent change^2^
**Overall intervention effect**	-	-	-	-	-	**0.21 [0.12, 0.30]**	-
*HIV knowledge score*							
6 weeks (n = 397)	5.9 (1.2)	5.9 (1.3)	5.9 (1.2)	-0.68	0.495	0.21 [0.12, 0.30]	0.1%
9 months (n = 387)	6.1 (1.3)	6.1 (1.3)	6.2 (1.3)	-0.70	0.485	0.23 [0.14, 0.32]	0.3%
*HIV treatment knowledge score*							
6 weeks (n = 397)	7.0 (0.8)	7.0 (0.8)	6.9 (0.8)	0.41	0.682	0.21 [0.12, 0.30]	0.2%
9 months (n = 387)	7.2 (0.7)	7.2 (0.7)	7.2 (0.7)	0.52	0.603	0.23 [0.14, 0.32]	0.1%
*ART medication beliefs score*							
6 weeks (n = 397)	4.1 (0.6)	4.0 (0.6)	4.1 (0.6)	-0.62	0.535	0.21 [0.12, 0.30]	0.1%
9 months (n = 386)	4.0 (0.6)	3.9 (0.5)	4.0 (0.6)	-1.95	0.052	0.23 [0.14, 0.32]	0.9%
*Adherence self-efficacy score*							
6 months (n = 394)	4.7 (0.4)	4.7 (0.4)	4.7 (0.4)	-0.49	0.628	0.25 [0.16, 0.33]	4.3%
12 months (n = 383)	4.6 (0.5)	4.7 (0.5)	4.6 (0.6)	1.70	0.090	0.20 [0.10, 0.29]	-2.1%
*HIV-related stigma score*							
6 weeks (n = 397)	2.2 (0.7)	2.2 (0.7)	2.1 (0.7)	1.50	0.135	0.21 [0.12, 0.30]	-1.1%
12 months (n = 392)	2.3 (0.6)	2.3 (0.7)	2.3 (0.6)	-0.75	0.454	0.20 [0.11, 0.29]	-1.2%
*Social support score*							
6 months (n = 394)	4.3 (0.8)	4.3 (0.8)	4.3 (0.8)	-1.10	0.273	0.24 [0.15, 0.33]	3.3%
12 months (n = 391)	4.5 (0.7)	4.5 (0.7)	4.5 (0.7)	0.00	0.999	0.20 [0.11, 0.29]	0.4%
*Patient-provider relationship score*							
6 weeks (n = 397)	3.8 (0.3)	3.8 (0.3)	3.8 (0.3)	0.73	0.466	0.21 [0.12, 0.30]	-0.6%
6 months (n = 393)	3.6 (0.5)	3.7 (0.3)	3.5 (0.5)	4.14	< 0.001	0.24 [0.15, 0.33]	1.8%
9 months (n = 387)	3.6 (0.5)	3.6 (0.4)	3.5 (0.5)	2.72	0.006	0.23 [0.14, 0.32]	0.2%
12 months (n = 378)	3.5 (0.5)	3.6 (0.5)	3.4 (0.5)	2.75	0.006	0.22 [0.13, 0.31]	0.1%

Abbreviations: SD: standard deviation; 95% CI: 95% confidence interval; ART: antiretroviral therapy. ^1^ Statistic from Wilcoxon rank-sum test. ^2^ Percentage change in risk difference when each potential mediator is added to the model, where both models are restricted to women with data for the potential mediator.

**Table 5:** Associations between potential postpartum mediators and the primary trial outcome (engagement in HIV care and viral suppression at 12 months postpartum)

	Total sample – Mean (SD)	Achieved primary outcome – Mean (SD)	Did not achieve primary outcome – Mean (SD)	z statistic^1^	p-value
*HIV knowledge score*					
6 weeks (n = 397)	5.9 (1.2)	5.9 (1.3)	6.0 (1.2)	0.87	0.386
9 months (n = 387)	6.1 (1.3)	6.1 (1.3)	6.2 (1.3)	1.34	0.181
*HIV treatment knowledge score*					
6 weeks (n = 397)	7.0 (0.8)	7.0 (0.8)	6.9 (0.8)	-0.89	0.374
9 months (n = 387)	7.2 (0.7)	7.2 (0.7)	7.2 (0.8)	0.24	0.810
*ART medication beliefs score*					
6 weeks (n = 397)	4.1 (0.6)	4.1 (0.6)	4.1 (0.6)	0.09	0.930
9 months (n = 386)	4.0 (0.6)	3.9 (0.6)	4.0 (0.5)	0.62	0.535
*Adherence self-efficacy score*					
6 months (n = 394)	4.7 (0.4)	4.8 (0.4)	4.7 (0.5)	-1.69	0.092
12 months (n = 383)	4.6 (0.5)	4.6 (0.5)	4.6 (0.6)	-1.56	0.119
*HIV-related stigma score*					
6 weeks (n = 397)	2.2 (0.7)	2.2 (0.7)	2.1 (0.7)	-0.81	0.421
12 months (n = 392)	2.3 (0.6)	2.3 (0.6)	2.4 (0.7)	1.11	0.266
*Social support score*					
6 months (n = 394)	4.3 (0.8)	4.4 (0.8)	4.2 (0.8)	-2.17	0.030
12 months (n = 391)	4.5 (0.7)	4.5 (0.7)	4.4 (0.8)	-1.05	0.293
*Patient-provider relationship score*					
6 weeks (n = 397)	3.8 (0.3)	3.8 (0.3)	3.8 (0.3)	-0.30	0.767
6 months (n = 393)	3.6 (0.5)	3.6 (0.5)	3.6 (0.4)	-1.19	0.233
9 months (n = 387)	3.6 (0.5)	3.6 (0.5)	3.6 (0.5)	0.56	0.574
12 months (n = 378)	3.5 (0.5)	3.5 (0.5)	3.5 (0.5)	-0.12	0.904

Abbreviations: SD: standard deviation; ART: antiretroviral therapy. ^1^ Statistic from Wilcoxon rank-sum test.

## Discussion

Using data from the MCH-ART study, which showed that integrated maternal and child health care during the postpartum period significantly increased retention in HIV care and viral suppression [12], this analysis examined psychosocial modifiers and mediators of the intervention effect. Here, we found evidence to suggest that the intervention was more effective among women experiencing higher levels of poverty, an unintended pregnancy, and higher levels of HIV-related stigma, while the intervention was less effective among women reporting risky drinking. None of the psychosocial constructs assessed mediated the association between trial arm and the primary outcome. Compared to women allocated to the standard of care, women allocated to the intervention reported significantly better relationships with their healthcare providers through 12 months postpartum, but better patient-provider relationships did not lead to improved outcomes.

Taken together, the intervention appeared to be most effective among women experiencing added vulnerability due to poverty, unintended pregnancy, and HIV-related stigma. We have previously demonstrated that unintended pregnancy is associated with elevated VL during the postpartum period [26]. Here, these data suggest that integrated care may be more beneficial for women reporting an unintended, compared to an intended, pregnancy. Unintended pregnancy is associated with delayed initiation of antenatal care [59–61], and consequently ART initiation later in pregnancy, and typically occurs in the context of multiple other vulnerabilities [62]. Consistent with the findings of this analysis, we observed stronger intervention effects among women initiating ART later in pregnancy, compared to those initiating earlier, as part of the primary trial analysis [12]. We hypothesise that the intervention may have provided a foundation for establishing and maintaining ART adherence. Remaining in the integrated service after delivery and having additional time during which to establish positive ART behaviours may have been more beneficial for women who had entered antenatal care later, compared to women who had entered care earlier and who had had more time to establish these behaviours prior to delivery [12].

Our finding regarding alcohol use is of particular concern. In South Africa, alcohol use during pregnancy is a major public health problem. High levels of problematic alcohol use have been observed among women living both with and without HIV in this setting [63–67], and substance use itself is a risk factor for poor ART outcomes [19, 24, 67–70]. Notably, alcohol use modified the intervention effect in this trial, with the intervention not associated with retention in HIV care and viral suppression among women reporting risky drinking in the 12 months prior to pregnancy recognition. Given that 26% of women in this study reported risky drinking during this time period, this is a large, high-risk group that warrants further attention. Psychological interventions have shown some effectiveness in promoting abstinence or reducing consumption among pregnant and postpartum women [71], and may be effective in reducing alcohol use and improving ART adherence among non-pregnant women living with HIV in South Africa [72]. Our findings suggest that more intensive interventions may be needed in place of or in conjunction with integrated HIV care to improve ART outcomes among pregnant women reporting a history of risky drinking.

In this secondary analysis, we observed no single definitive mediator of the intervention effect. We hypothesise that the intervention may be effective through a range of mechanisms that may operate differently within different groups of women, and should be explored in a larger trial. For example, integrated MCH services reduce the economic burden of attending separate visits and may thus be of most benefit to women experiencing higher levels of poverty [73]. In addition, we hypothesise that receiving HIV care concurrently with infant health care within the MCH clinic may lead to less inadvertent disclosure than attending a separate adult HIV clinic, and thus may be most beneficial to women who experience the highest levels of stigma [12]. However, care must be taken to avoid inadvertent HIV-status disclosure when providing integrated PMTCT and MCH services [13]. Although the MCH-ART trial was conducted in a single clinic, these findings may be of relevance to all contexts where levels of poverty and stigma are high. In this study, women allocated to the intervention reported significantly better relationships with their healthcare providers. Although we did not observe an association between better patient-provider relationships and improved engagement in care and viral suppression, continuity of care with the same provider may have other positive effects. However, these effects may not persist after transfer out of integrated services to new healthcare providers, and further work is needed to support women after the point of transfer [74, 75].

These findings should be considered in light of several limitations. They arise from a single antenatal clinic in a peri-urban area of South Africa and only women who were initiating ART during pregnancy were enrolled into the trial, thus the findings may not be generalisable to all postpartum women living with HIV. Although all postpartum women may benefit from integrated HIV and MCH care, only breastfeeding women were enrolled into the trial, thus limiting generalisability. Pregnant women were enrolled into the trial between March 2013 and April 2014 and were followed through January 2016, and changes in PMTCT and HIV care since that time may make our findings less generalisable to the current context. The psychosocial constructs explored were self-reported and are subject to recall and social desirability bias, which may be particularly relevant for behaviours such as alcohol use, although we used validated scales wherever possible. However, it is plausible that we did not observe hypothesised mediators of the intervention effect due to the challenges of measuring complex constructs, with the measures administered potentially not accurately representing the construct of interest. Although these measures were originally developed several decades ago in high-income countries, the constructs assessed and items included in these scales are still relevant and applicable to multiple settings. Although we attempted to collect patient-level data on direct and indirect costs to measure the economic burden of attending clinic visits, these data were only available for a subset of participants and it was not possible to accurately quantify costs at the individual level [73]. In addition, true modifiers and mediators of the intervention effect may not have been measured as part of the trial. As noted above, all analyses should be considered exploratory. The small number of women in some psychosocial subgroups led to wide confidence intervals when examining potential modifiers of the intervention effect and may have reduced our power to detect significant differences, while also limiting our ability to examine mechanisms within different subgroups.

## Conclusions

Despite these limitations, this analysis provides novel and important findings related to modifiers and mediators of the effectiveness of integrated care for postpartum women living with HIV. Our findings suggest that integrated services are associated with significantly better patient-provider relationships over time. Further, women who face additional vulnerabilities due to poverty and unintended pregnancy may benefit the most from integrated care, and integrated services could potentially be targeted to settings where these characteristics are particularly prevalent. Notably, the benefits of integrated care appear to be hampered by alcohol use, suggesting that women experiencing problematic alcohol use warrant further attention in intervention development and evaluation.

### Electronic supplementary material

Supplementary material 1

## Data Availability

All relevant data are within the paper.

## Code Availability

N/A.
