# Supplementary material for: Why do Integrated Maternal HIV and Infant Healthcare Services work? A Secondary Analysis of a Randomised Controlled Trial in South Africa
Source: AIDS Behav. 2023 Jun 12;27(12):3831–43. doi: 10.1007/s10461-023-04097-x (PMC10598190; doi:10.1007/s10461-023-04097-x)
Supplement: Supplementary file 1 — Supplementary material 1 [file 10461_2023_4097_MOESM1_ESM.docx]

| **Supplementary Table 1** Comparison of women enrolled into the randomised controlled trial versus those not enrolled | | | | | |
| --- | --- | --- | --- | --- | --- |
|  | All women enrolled into antenatal follow-up – Mean (SD) | Enrolled into the trial  – Mean (SD) | Not enrolled into the trial  – Mean (SD) | χ^2^ or z statistic^1^ | P-value |
| Number of participants | 628 | 471 | 157 |  |  |
| Age in years | 28.6 (5.4) | 28.6 (5.4) | 28.8 (5.2) | 0.59 | 0.553 |
| N (%) having completed secondary/any tertiary education | 166 (26%) | 117 (25%) | 49 (31%) | 2.46 | 0.117 |
| N (%) currently employed | 238 (38%) | 184 (39%) | 54 (34%) | 1.09 | 0.296 |
| N (%) married and/or cohabiting | 259 (41%) | 193 (41%) | 66 (42%) | 0.05 | 0.815 |
| N (%) diagnosed HIV-positive during current pregnancy | 345 (55%) | 268 (57%) | 77 (49%) | 2.94 | 0.087 |
| Gestation at entry into antenatal care (weeks) | 20.5 (7.5) | 20.9 (7.6) | 19.3 (6.9) | -2.36 | 0.019 |
| Poverty categories – n (%)  Most disadvantaged  Moderate disadvantage  Least disadvantaged | 227 (36%)  208 (33%)  193 (31%) | 164 (35%)  158 (34%)  149 (32%) | 63 (40%)  50 (32%)  44 (28%) | 1.52 | 0.468 |
| N (%) reporting an unintended pregnancy | 442 (70%) | 338 (72%) | 104 (66%) | 1.72 | 0.190 |
| HIV knowledge score (max=9; n=625) | 5.9 (1.4) | 5.9 (1.4) | 5.9 (1.4) | 0.18 | 0.859 |
| HIV treatment knowledge score (max=8; n=625) | 6.5 (1.2) | 6.5 (1.2) | 6.6 (1.2) | 1.93 | 0.053 |
| ART medication beliefs score (max=5; n=625) | 4.0 (0.7) | 4.1 (0.7) | 4.0 (0.7) | -0.53 | 0.593 |
| Adherence self-efficacy score – 2^nd^ antenatal visit (max=5; n=623) | 4.8 (0.4) | 4.8 (0.4) | 4.8 (0.5) | 0.47 | 0.638 |
| Adherence self-efficacy score – late 3^rd^ trimester (max=5; n=598) | 4.8 (0.4) | 4.8 (0.4) | 4.8 (0.4) | 0.17 | 0.862 |
| HIV-related stigma score (max=5; n=624) | 2.2 (0.8) | 2.2 (0.8) | 2.1 (0.9) | -0.73 | 0.464 |
| Social support score – 2^nd^ antenatal visit (max=5; n=625) | 4.2 (0.8) | 4.2 (0.8) | 4.4 (0.8) | 1.83 | 0.068 |
| Social support score – late 3^rd^ trimester (max=5; n=599) | 4.3 (0.8) | 4.3 (0.8) | 4.3 (0.8) | -0.82 | 0.413 |
| N (%) scoring above threshold for depression (EPDS; n=625) | 68 (11%) | 48 (10%) | 20 (13%) | 0.81 | 0.369 |
| N (%) scoring above threshold for psychological distress (K-10; n=625) | 35 (6%) | 27 (6%) | 8 (5%) | 0.09 | 0.767 |
| N (%) scoring above threshold for risky drinking – 2^nd^ antenatal visit (AUDIT-C; n=625) | 163 (26%) | 118 (25%) | 45 (29%) | 0.83 | 0.364 |
| N (%) scoring above threshold for risky drinking – late 3^rd^ trimester (AUDIT-C; n=599) | 58 (10%) | 43 (9%) | 15 (11%) | 0.45 | 0.502 |
| N (%) reporting any intimate partner violence – 2^nd^ antenatal visit (n=623) | 132 (21%) | 105 (22%) | 27 (17%) | 1.88 | 0.171 |
| N (%) reporting any intimate partner violence – <7 days postpartum (n=597) | 36 (6%) | 32 (7%) | 4 (3%) | 2.30 | 0.145 |
| Abbreviations: SD: standard deviation; EPDS: Edinburgh Postnatal Depression Scale; K-10: Kessler-10 scale; AUDIT-C: Alcohol Use Disorders Identification Test – Consumption. All measures are from the 2^nd^ antenatal visit, unless otherwise specified. ^1^ Statistic from χ^2^ or Wilcoxon rank-sum test. | | | | | |
